# Effects of Varying Gravity Levels in Parabolic Flight on the Size-Mass Illusion

**DOI:** 10.1371/journal.pone.0099188

**Published:** 2014-06-05

**Authors:** Gilles Clément

**Affiliations:** International Space University, Illkirch-Graffenstaden, France; Ludwig-Maximilian University, Germany

## Abstract

When an observer lifts two objects with the same weight but different sizes, the smaller object is consistently reported to feel heavier than the larger object even after repeated trials. Here we explored the effect of reduced and increased gravity on this perceptual size-mass illusion. Experiments were performed on board the CNES Airbus A300 Zero-G during parabolic flights eliciting repeated exposures to short periods of zero g, 0.16 g, 0.38 g, one g, and 1.8 g. Subjects were asked to assess perceived heaviness by actively oscillating objects with various sizes and masses. The results showed that a perceptual size-mass illusion was clearly present at all gravity levels. During the oscillations, the peak arm acceleration varied as a function of the gravity level, irrespective of the mass and size of the objects. In other words we did not observe a sensorimotor size-mass illusion. These findings confirm dissociation between the sensorimotor and perceptual systems for determining object mass. In addition, they suggest that astronauts on the Moon or Mars with the eyes closed will be able to accurately determine the relative difference in mass between objects.

## Introduction

After lifting two objects with identical mass but different sizes, participants invariably report that the small object feels heavier than the large object. This perceptual size-mass illusion has been studied since the 19^th^ century with the aim of understanding how relevant dimensions of a physical stimulus are scaled by the human perceptual system to produce experience of *heaviness* [1], [2]. Many theories have been proposed, in attempts to ascertain the underlying causes of this misperception of mass, but they remain controversial at this point [3]. One hypothesis, the so-called *sensory mismatch hypothesis* [4], is that the smaller object is judged to be heavier than the larger object because the proprioceptive sensory feedback received during lifting does not match the predicted sensory feedback generated by the efferent copy of the motor command. The large object is represented as “light” because it is lighter than expected, and the small object is represented as “heavy” because it is heavier than expected. This initial perception does not go away or even diminish after repeated lifting attempts, despite the fact that the feedback mismatch diminishes quickly as the sensorimotor system adapts by decreasing the force or force rate used to lift the larger object and increasing the force or force rate used to lift the smaller object [5]. Therefore, another hypothesis is that perception and action use separate representations of the object’s mass. On one hand, the perception of the object’s mass would be inherently *relative* and driven by characteristics that are more useful for cognitive processing. On the other hand the sensorimotor (action) processing of the object’s mass would be *absolute* and driven by characteristics that are more necessary for accurate control of action [6].

On Earth both the weight and the mass of objects can be sensed, whereas in the absence of gravitational forces, such as in Earth orbit, weight cues are effectively absent. In weightlessness (zero g), mass can only be evaluated via inertial cues by accelerating (shaking) the objects. The primary objective of this experiment was to explore the relationship between perceptual and sensorimotor systems in altered gravity environments during parabolic flight using the *size-mass illusion* (SMI). Previous ground-based studies have investigated the SMI by suspending objects from short strings [8] or a long pair of wires [9], [10] and by asking the subject to push the objects and rate their heaviness. In a recent study subjects were instructed to move objects with different mass and size back and forth on a horizontal air bearing slide with negligible friction [11]. These studies concluded that a SMI occurred independent of the contribution of gravity. Based on these results, our hypothesis was that the SMI should have the same magnitude in one g as in Mars and Moon gravity, or even in micro- or hypergravity.

Previous experiments performed in orbital and parabolic flight have shown that people are able to use inertial cues to discriminate the mass of objects by shaking them back and forth, although they are not as accurate as in a situation in which they have also gravitational cues available [12], [13]. However, these experiments do not tell us whether the size-mass illusion is still present in weightlessness, as the objects used all had the same size. In the present experiment we investigated in parabolic flight the magnitude of the SMI in which the subjects shook objects with the same mass but different heights before making verbal judgments of heaviness. We also investigated the subjects’ ability to accurately estimate the mass of objects with same size and different mass as controls, and compared their responses with the eyes open and the eyes closed. These controls were lacking in the previous ground-based studies [8]–[11]. Measurements of arm acceleration during shaking were also performed to control that arm movements were not different between objects with the same mass.

## Materials and Methods

### Ethics Statement

These experiments were undertaken with the understanding and written consent of each subject. The test procedures were approved by the European Space Agency medical board and by the Comité de Protection des Personnes Nord Ouest III (Caen, France) and were performed in accordance with the ethical standards laid down in the 1964 Declaration of Helsinki.

### Equipment

This study used objects composed of 2.54-cm hollow plastic cubes (Table 1). To vary the mass, some of the plastic cubes were filled with sand (Experiment 1 and 2) or with 5-mm steel balls (Experiment 3). Care was taken to ensure that the center of mass of all objects was coincident with their centroid. To vary the size, some objects were made taller but their width and depth were kept constant so that the subject handgrip was the same for all objects. The objects were wrapped with a white, glossy adhesive sheet so that they all had uniform appearance except for their height.

**Table 1:** Characteristics of the objects used in the three experiments.

Experiments 1 and 2							
Object		1	2	3		4	5
Mass, gr		265 (75)	308 (87)	**354 (100)**		354 (100)	354 (100)
Height, cm		7.6 (100)	7.6 (100)	**7.6 (100)**		12.7 (166)	15.2 (200)
Width, cm		7.6 (100)	7.6 (100)	**7.6 (100)**		7.6 (100)	7.6 (100)
Depth, cm		7.6 (100)	7.6 (100)	**7.6 (100**)		7.6 (100)	7.6 (100)
Volume, cm^3^		442 (100)	442 (100)	**442 (100)**		737 (166)	885 (200)
Density, gr/cm^3^		.060 (75)	0.69 (87)	**0.80 (100)**		0.48 (60)	0.40 (50)
**Experiment 3**							
**Object**	**1**	**2**	**3**	**4**	**5**	**6**	**7**
Mass, gr	500 (50)	750 (75)	875 (87)	**1000 (100)**	1000 (100)	1000 (100)	1000 (100)
Height, cm	7.6 (100)	7.6 (100)	7.6 (100)	**7.6 (100)**	10.2 (133)	12.7 (166)	15.2 (200)
Width, cm	7.6 (100)	7.6 (100)	7.6 (100)	**7.6 (100)**	7.6 (100)	7.6 (100)	7.6 (100)
Depth, cm	7.6 (100)	7.6 (100)	7.6 (100)	**7.6 (100)**	7.6 (100)	7.6 (100)	7.6 (100)
Volume, cm^3^	442 (100)	442 (100)	442 (100)	**442 (100)**	590 (133)	737 (166)	885 (200)
Density, gr/cm^3^	1.13 (50)	1.69 (75)	1.98 (87)	**2.26 (100)**	1.69 (75)	1.36 (60)	1.13 (50)

Numbers in parentheses are in percent relative to the reference cube (columns in bold). The objects in Experiment 3 were made heavier than those in Experiments 1 and 2 for the tests in zero g.

### Experiment 1– Comparison between Size-weight and Size Mass Illusions

This experiment was performed in the laboratory. Twelve subjects (6 female, 6 male), ranging in age from 22–56 years (mean 32.4 years) participated in this study. The subjects sat in front of table on which there was a smooth plastic mat. The surface of the mat was sprayed with a dry lubricant to reduce friction. By measuring the applied force to move the various objects on the mat the coefficients of static and dynamic friction were evaluated to be 0.27 and 0.11, respectively. For mass perception, the subjects oscillated the objects side to side by performing horizontal flexion/extension of the elbow while looking at the objects, for five seconds. The subjects were instructed to repeatedly move the objects from one end to the other of the mat (amplitude 40 cm), but no instruction was given regarding the frequency or speed of arm motion. After each oscillation period, the subjects were asked to estimate the mass of the object using a scale from 1 to 10 (1 being light and 10 being heavy) according to the procedure described by Grandy & Westwood [5].

For weight perception, the subject extended his/her dominant hand palm up. The operator placed the object in the subject’s hand. The subject held his arm and wrist still and constantly looked at the object for five seconds. After each holding period, the subjects was asked to estimate the weight of the object using a scale from 1 to 10 (1 being light and 10 being heavy) according to the procedure described by Grandy & Westwood [5].

This experiment used five objects: three objects had the same mass but a different height (100%, 166%, and 200%); and three cubes had the same height but a different mass (100%, 87% and 75%) (Table 1). Each object was presented five times in random order.

### Experiment 2– Comparison between One g, 0.16 g and 0.38 g

This experiment took place in December 2012 on board the Airbus A-300 Zero-G during the second CNES/ESA/DLR campaign of parabolic flight in Moon (0.16 g) and Mars (0.38 g) gravity. Each of the three flights lasted two to three hours and included 31 parabolic manoeuvres, i.e. 13 parabolas at 0.16 g, 12 at 0.38 g, and 6 at zero g in that order. Each parabola started with a pull-up phase and ended with a pull-out phase at 1.8 g, both lasting about 20 sec. The duration of the reduced gravity periods depended on the gravity level: about 21 sec for weightlessness, 24 sec for lunar gravity and 33 sec for Mars gravity.

Six subjects (one female, five male), ranging in age from 25–56 years (mean 47.0 years) participated in this experiment. All subjects had passed the equivalent of an Air Force Class III medical examination, and had normal or corrected-to-normal vision with no known visual deficits. Four subjects took prophylactic medication (a combination of promethazine and dexedrine) before boarding the plane, and none of them showed symptoms of motion sickness during the flight. Data were collected during three parabolas at 0.16 g and three parabolas at 0.38 g. Controls in one g were also performed on board the aircraft during straight and level flight between successive parabolas, while the medicated subjects were under the influence of the drug. This was to ensure that the changes seen across the various gravity levels were not due to the effect of the medication.

This experiment used the same objects as in Experiment 1 (Table 1). Each object was presented twice in random order. During testing, subjects sat on the aircraft floor, and oscillated the objects side to side between two markers 40 cm apart, while looking at them, for five seconds. After each oscillation period, the subjects were asked to estimate the mass of the object using a scale from 1 to 10 (1 being light and 10 being heavy) according to the procedure described by Grandy & Westwood [5]. A three-axis accelerometer (Gulf Coast Data Concept, LLC, Waveland, MS, USA) mounted on a wristband continuously recorded the subjects arm movement, for off-line calculation of the peak acceleration of subject’s arm motion at each of the gravity level.

### Experiment 3– Comparison between One g, Zero g, and 1.8 g

This experiment was performed during the June 2013 ESA campaign of parabolic flight when the airplane was flying only zero g parabolas. Testing was performed during the 20-sec 1.8 g pull-up phase, during the 20-sec zero g phase, and during one g periods when the aircraft was flying straight and level. Twelve subjects (two female, ten male), ranging in age from 19–57 years (mean 33.3 years) participated in this study. Eleven subjects took prophylactic medication before boarding the plane, and only two of them showed symptoms of motion sickness during the flight.

Prior to this campaign, a pilot study had indicated that the mass of the objects used in Experiments 1 and 2 were too small for reliable testing in zero g. Therefore, another set of objects was built for this experiment. The mass of the seven objects used in Experiment 3 ranged from 500 gr to 1000 gr. Four objects had the same mass but a different height (100%, 133%, 166%, and 200%); and four cubes had the same size but a different mass (100%, 87%, 75%, and 50%) (Table 1). Each object was presented four times in a random order.

Subjects sat in a standard aircraft seat with a lap desk attached across their thighs. The subjects oscillated the objects side to side within the limit of the lap desk, e.g. 40 cm, while looking at them, for five seconds. After each oscillation period, the subject was asked to estimate the mass of the object using a scale from 1 to 10 (1 being light and 10 being heavy). A three-axis accelerometer mounted on a wristband continuously recorded the subject’s arm movements.

Each subjects was tested during 15 parabolas. In addition, during the last two parabolas, each subject was asked to estimate the objects’ mass after oscillating them with the eyes closed. For this part of the experiment each object was tested only once. The objective was to confirm that the subject’s judgments of mass were not different for those objects that had a different height but the same mass.

### Data Analysis

Peak-to-peak acceleration of the arm in the horizontal plane was measured and averaged for three cycles of oscillations. The averaged value was then divided by two to obtain peak arm acceleration.

The mean frequency of arm oscillations was also calculated in each condition. Because the amplitude of arm movements was relatively constant (40 cm) their frequency varied as the peak arm acceleration. Therefore, only the measurements of the peak arm acceleration are reported in this paper.

Subjects’ judgment of heaviness and peak arm acceleration were analyzed with repeated measures ANOVAs in Excel. Using an alpha error of 0.05 as the decision rule, the null hypothesis was that there is no difference across gravity level and object size or mass.

## Results

### Experiment 1– Comparison between Size-weight and Size-mass Illusion

**Figure 1:**
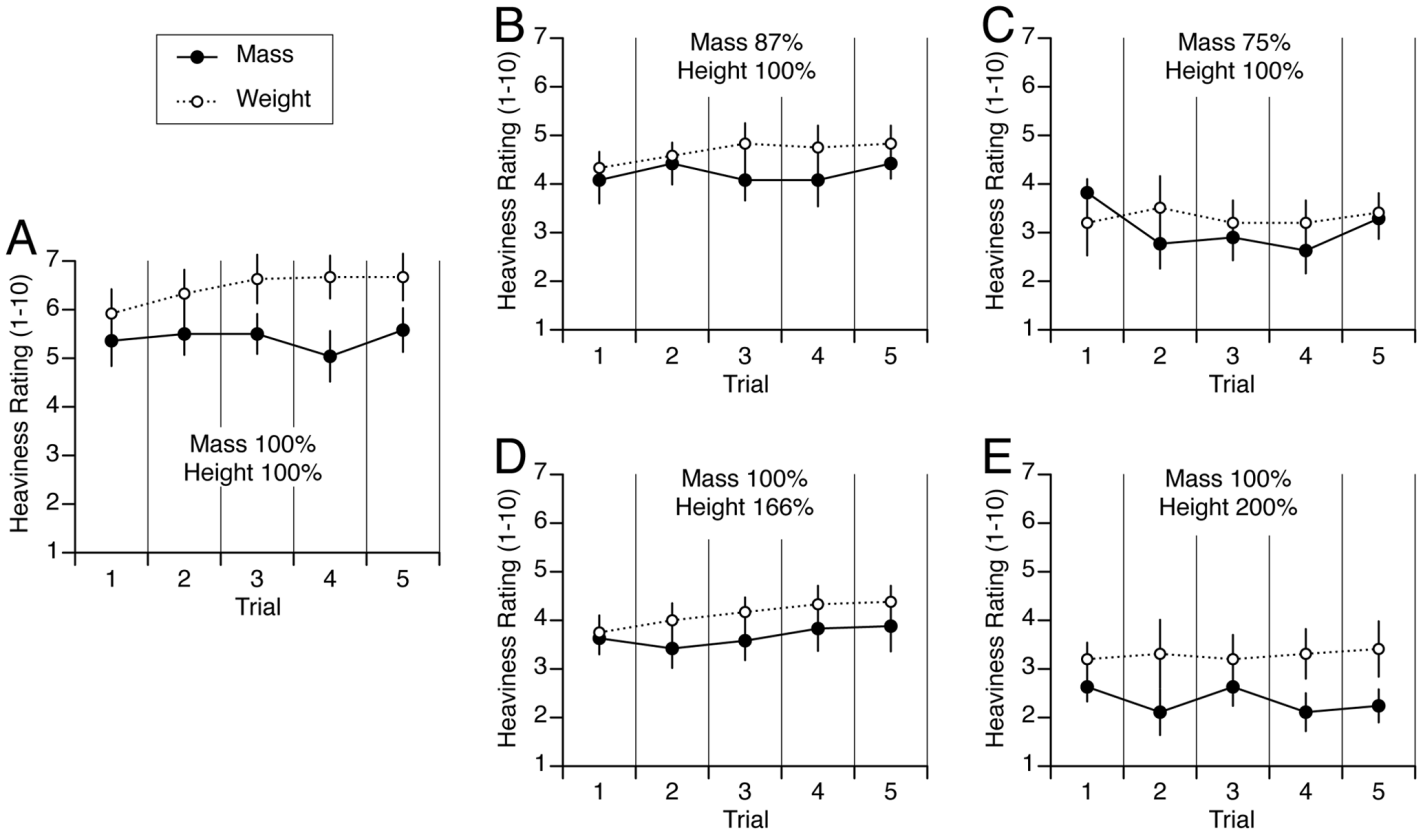
Comparison between heaviness rating of mass and weight for the five trials (mean ± SE of 12 subjects) with the five objects used in Experiment 1. Each graph represents the heaviness ratings of mass and weight for one object. In Figure **1A**, the object had a mass of 354 gr and a height of 7.6 cm. This object referred to as the reference cube (mass 100%, height 100%). In Figure **1B** and **1C**, the objects had the same height (100%) but a smaller mass (87%, and 75%, respectively). In Figure **1D **and **1E**, the objects had the same mass (100%) but a larger height (12.7 and 15.2 cm, or 166% and 200% respectively). Except for the object shown in Fig. 1C, for all the other objects the heaviness ratings was significantly different between mass and weight (one-way ANOVA, p<0.01).

Verbal judgments of heaviness were analyzed using a 5 (objects)×2 (estimate methods; mass vs. weight estimates) repeated-measures ANOVA, alpha = 0.05. There was a main significant effect of object [F (4,40) = 223.1, p<0.001], a significant effect of estimates method [F(1,40) = 73.12, p<0.001], as well as a significant effect of interaction [F (4,40) = 4.71, p = 0.003]. This indicates that the mass and weight estimates were significantly different across the objects and methods. Except for the object shown in C, for all the other four objects the weight estimates were significantly larger than the mass estimates (paired t-test, p<0.01) (Figure 1). These results are in agreement with those of previous studies showing that weight discrimination is better than inertia discrimination [8], [9], [13].

**Figure 2:**
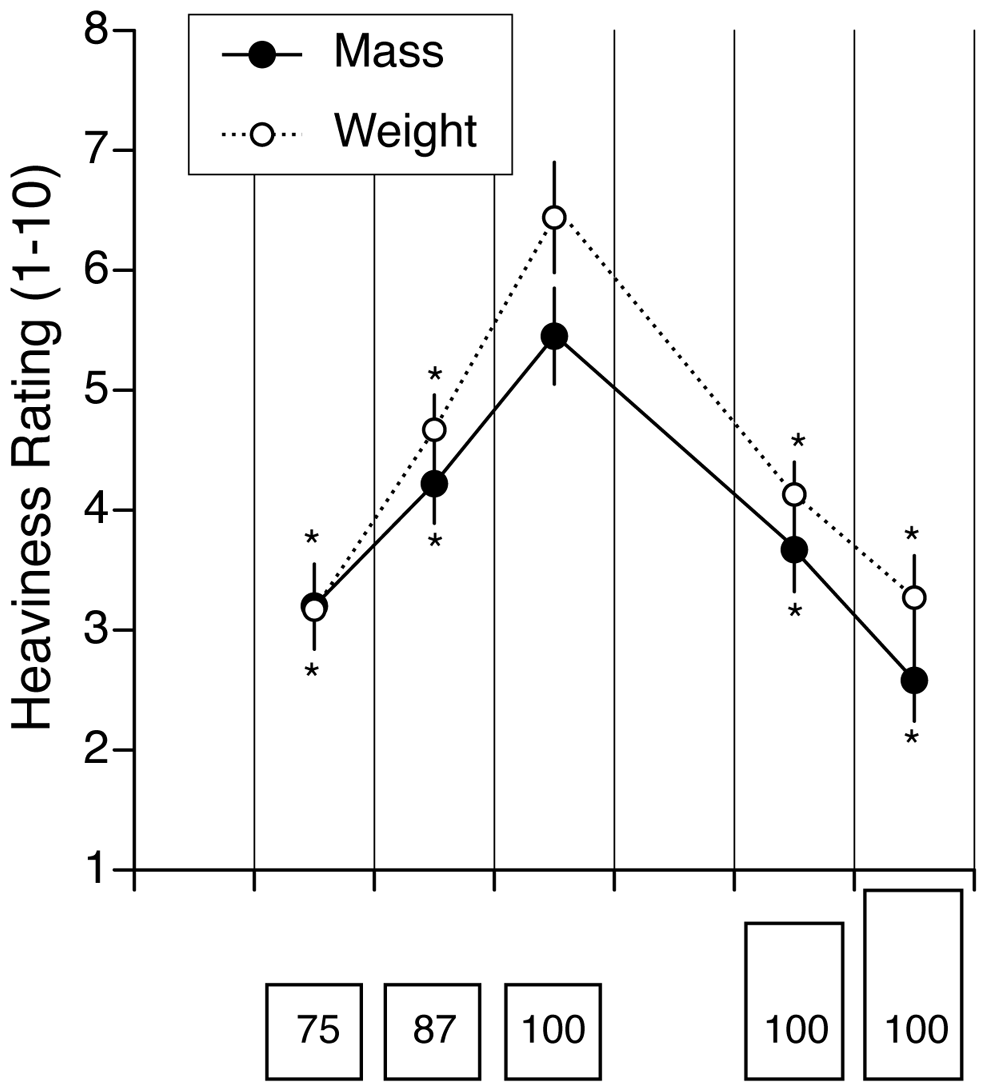
Comparison between the heaviness ratings for the mass and weight estimation (mean ± SE of five trials with 12 subjects). *p<0.01. Compared with the reference cube (100% mass) the objects with a taller height were systematically perceived as being lighter.

Verbal judgments of weight and mass were then analyzed separately for the three objects with the same height but a different mass (Figure 1A, 1D, 1E) and for the three objects with the same mass but a different height (Figure 1A, 1B, 1C). Using a 5 (trials)×3 (objects) repeated-measures ANOVA, a significant main effect of object size indicated that participants perceived the weight [F (2,165) = 48.89, p<0.001] and mass [F (2,165) = 51.2, p<0.001] of the small object to be heavier than the large object throughout the entire experiment, despite the fact that the small object actually had less mass (Figure 2). No significant main effect of trials indicated that the perceptual reports of weight [F (4,165) = 1.08, p = 0.36] and mass [F (4,165) = 0.40, p = 0.80] did not change significantly over the five trials. Importantly, no significant interaction was found between trials and object indicating that the magnitude of the perceptual SMI [F (8,165) = 0.15, p = 0.99] and SMI [F (8,165) = 0.71, p = 0.78] was unchanged during the course of the experiment. This result is in agreement with previous studies that showed that the SMI only habituates after multiday practice and several hundreds of trials [14].

Not surprisingly, for the three objects with the same height but a different mass, there was a significant effect of object mass on the judgment of weight [F (2,165) = 20.90, p<0.001] and the judgment of mass [F (2,165) = 18.84, p<0.001]. The lighter the object, the lighter the perceived weight and mass (Figure 2). No significant main effect of trials indicated that the perceptual reports of weight [F (4,165) = 0.64, p = 0.63] and mass [F (4,165) = 1.11, p = 0.35] did not change significantly over the five trials.

### Experiment 2– Comparison between One g, 0.16 g and 0.38 g

**Figure 3:**
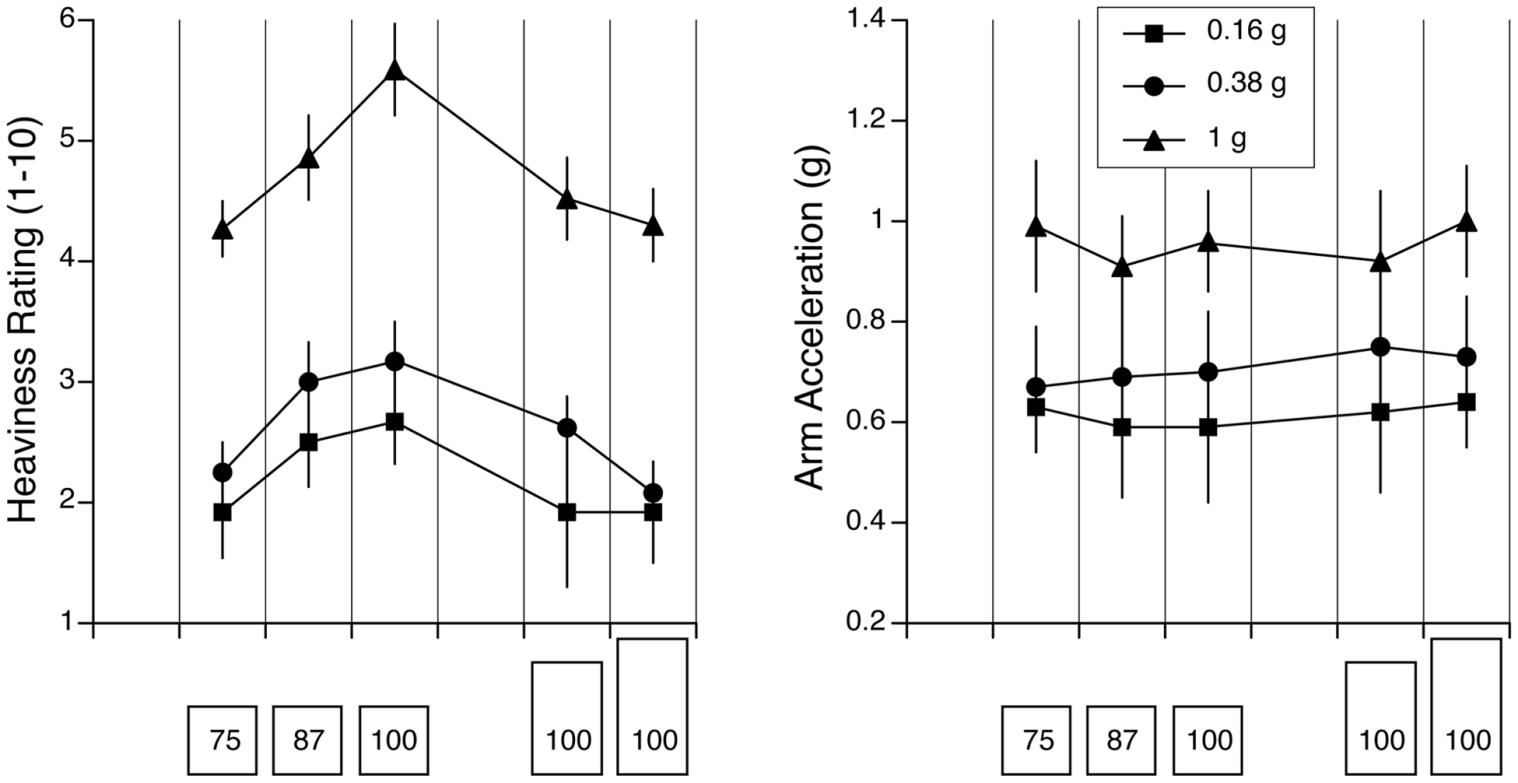
Heaviness ratings (A) and peak arm acceleration (B) for the five objects tested in 0.16 g, 0.38 g, and one g (mean ± SE of two trials with six subjects). The mass estimates and arm accelerations were significantly different across gravity levels. Compared to the reference cube, the heaviness ratings were significantly smaller for the taller and the lighter objects. However, the arm acceleration was not different between the five objects.

Verbal judgments of heaviness were analyzed using a 3 (objects)×3 (gravity; one g, 0.16 g, 0.38 g) repeated-measures ANOVA. Because no significant differences were observed across trials in Experiment 1, the individual responses to the successive trials with the same objects were averaged together. For the three objects that had the same mass but a different height, a two-way ANOVA yielded a main effect for object’s height [F (2,45) = 3.37, p = 0.04], such that the average subjects’ judgments of mass were significantly lower for objects 166% taller (M = 3.42) and 200% taller (M = 2.97) than the reference cube (M = 3.67). The effect of gravity level was also significant [F (2,45) = 40.3, p<0.001]. Subjects’ judgments of mass were lower at 0.16 g (M = 2.36) and 0.38 g (M = 3.03) compared to one g (M = 4.67) (Figure 3). The interaction effect was non-significant [F (4,45) = 0.66, p = 0.62], indicating that the SMI was not significantly affected by the gravity level.

There was no effect of object height on the arm accelerations during the mass estimation tests [F (2,45) = 0.39, p = 0.68], thus indicating the absence of a sensorimotor equivalent of the SMI. The peak arm accelerations were not significantly different for objects whose height was 166% (M = 0.77 g) or 200% (M = 0.79 g) taller than the reference cube (M = 0.75 g). However, there was a significant effect of gravity on the arm acceleration [F (2,45) = 26.31 p<0.001]. Arm accelerations were significantly lower at 0.16 g (M = 0.62 g) and 0.38 g (M = 0.73 g) compared to one g (M = 0.96 g) (Figure 3). The interaction effect between object size and gravity was non-significant [F (4,45) = 0.19, p = 0.93].

As expected, for the three cubes that had different mass, there was a main effect of object mass [F (2,45) = 7.75, p<0.001]. There was also a significant effect of gravity [F (2,45) = 52.66, p<0.001] but no significant effect interaction between the two factors. Subjects’ verbal judgments of heaviness were significantly lower at 0.16 g (M = 2.17) and 0.38 g (M = 2.50) than at one g (M = 4.63) (Figure 3).

For the three cubes, there was no significant effect of object mass on the arm accelerations. The peak arm acceleration was 0.75 g for the reference cube (mass 100%), 0.77 g for the 87%-mass cube, and 0.73 g for the 75%-mass cube. A significant main effect of gravity level on arm acceleration was found [F (3,45) = 27.88, p<0.001], but the interaction effect between object mass and gravity was non-significant [F (4,45) = 0.21, p = 0.92]. Arm accelerations were significantly lower at 0.16 g (M = 0.60 g) and 0.38 g (M = 0.69 g) compared to one g (M = 0.96 g) (Figure 3).

### Experiment 3– Comparison between One g, Zero g, and 1.8 g

**Figure 4:**
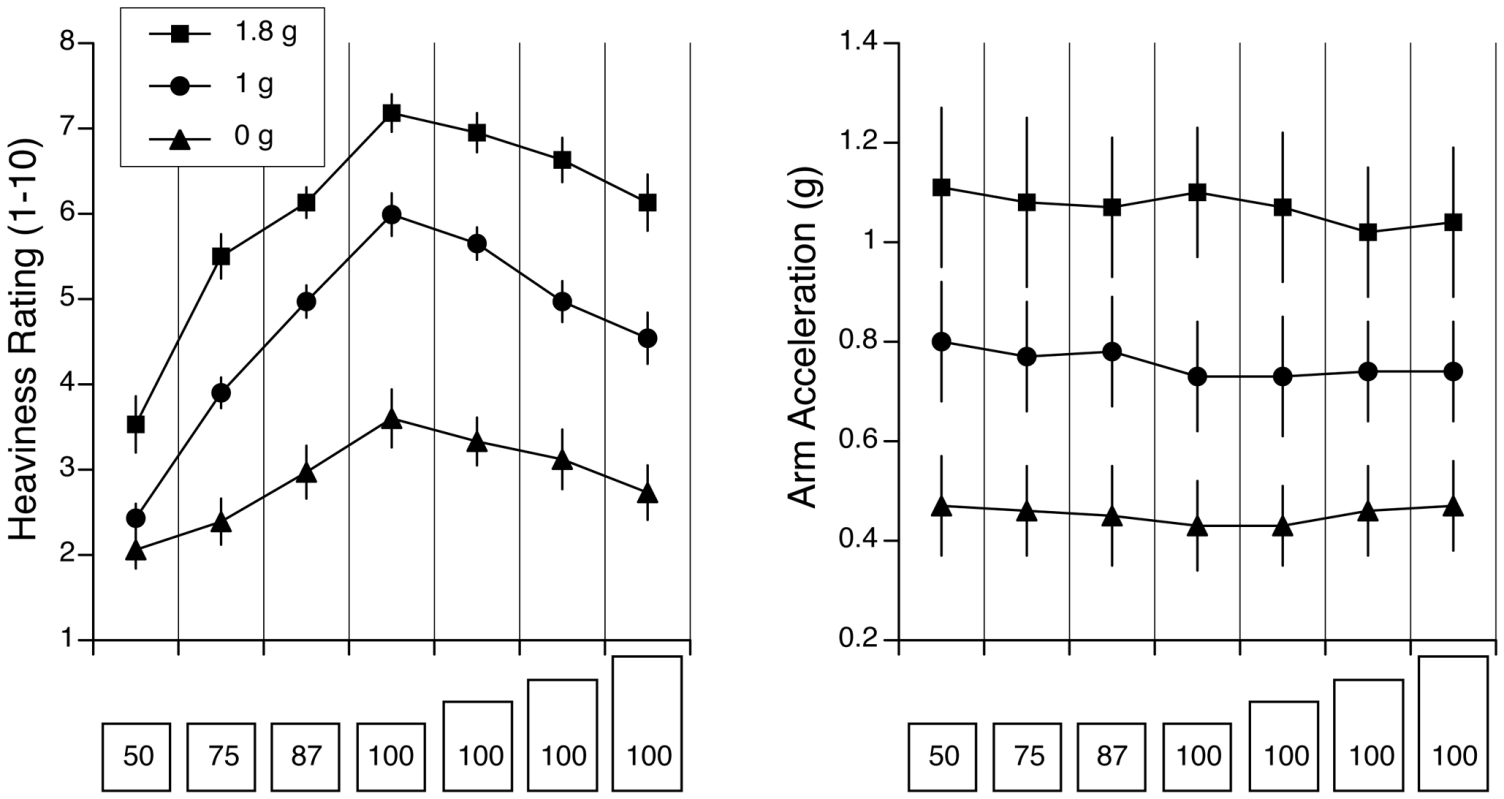
Heaviness ratings (A) and peak arm acceleration (B) for the five objects tested in zero g, one g, and 1.8 g (mean ± SE of four trials with 12 subjects). A size-mass illusion was present at all gravity levels, as shown by the smaller heaviness ratings for the taller objects compared to the reference cube. The arm acceleration varied as a function of the gravity level, but it was not different between the five objects.

Verbal judgments of heaviness were analyzed using a 4 (objects)×3 (gravity; one g, zero g, 1.8 g) repeated-measures ANOVA. For the four objects that had the same mass but a different height, a two-way ANOVA yielded a significant effect for object’s size [F (3,132) = 9.14, p<0.001], indicating that the SMI was present at both zero g and 1.8 g. There was also a significant effect of gravity level [F (2,132) = 160.1, p<0.001]. Subjects’ judgments of heaviness were lower at zero g (M = 3.19) and higher at 1.8 g (M = 6.73) than at one g (M = 5.29) (Figure 4). The interaction effect was non-significant [F (6,132) = 0.32, p = 0.92], thus indicating that the SMI was not significantly affected by the gravity level.

Arm accelerations during the mass estimation procedure were not significantly different when the object height was 133% (M = 0.75 g), 166% (M = 0.74 g) and 200% (M = 0.75 g) larger than the reference cube (M = 0.75 g). However, there was a significant effect of gravity [F (2,132) = 28.88 p<0.001]. Arm accelerations were higher at 1.8 g (M = 1.06 g), and lower at 0 g (M = 0.45 g) compared to one g (M = 0.74 g) (Figure 4). The interaction effect was non-significant [F (6,132) = 0.06, p = 0.99].

For the four cubes that had the same size but a different mass, there was a significant main effect of object mass [F (3,132) = 72.85, p<0.001] and gravity [F (2,132) = 128.5, p<0.001] and a significant effect of interaction between the two. Subjects’ judgments of mass were lower at zero g (M = 2.75) and higher at 1.8 g (M = 5.59) than at one g (M = 4.32) (Figure 4).

Peak arm accelerations were not significantly different when the object mass was 50% (M = .80 g), 75% (M = 0.77 g) and 87.5% (M = 0.77 g) that of the reference cube (M = 0.75 g). A significant main effect of gravity on arm acceleration was found [F (2,132) = 27.21, p<0.001], but the interaction effect was non-significant [F (6,132) = 0.02, p = 0.99]. Arm accelerations were significantly higher at 1.8 g (M = 1.09 g) and lower at zero g (M = 0.45 g) compared to one g (M = 0.77 g) (Figure 4).

**Figure 5:**
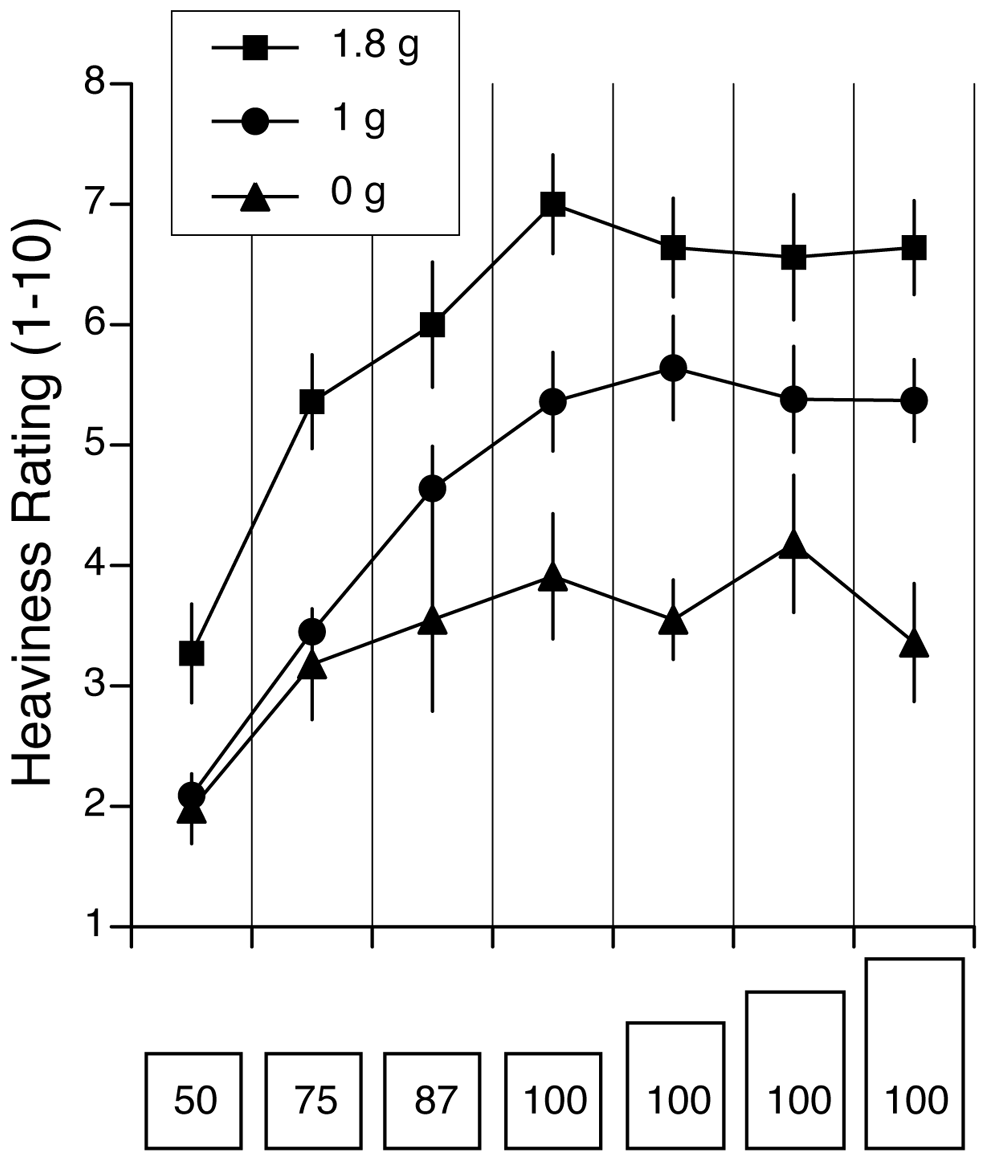
Heaviness ratings for the five objects tested in zero g, one g, and 1.8 g with the eyes closed (mean ± SE of one trial with 12 subjects). The mass estimates were the same for the four objects with the same mass and a different height, indicating that the size-mass illusion was no longer present.

As expected, with the eyes closed there was no significant effect of object size on perceived mass [F (3,132) = 0.33, p = 0.79]. There was a significant effect of gravity, however [F (2,132) = 51.29, p<0.001], but no interaction effect between object size and gravity [F (6,132) = 0.55, p = 0.76]. Similarly, and just like for the condition with the eyes open, there was no effect of size [F (2,132) = 0.05, p = 0.98], but an effect of gravity [F (2,132) = 15.37, p<0.001] on arm acceleration. The arm acceleration and the perception of mass responses obtained with the cubes of different mass were not different from those obtained with the eyes open (Figure 5).

### Combined Results

**Figure 6:**
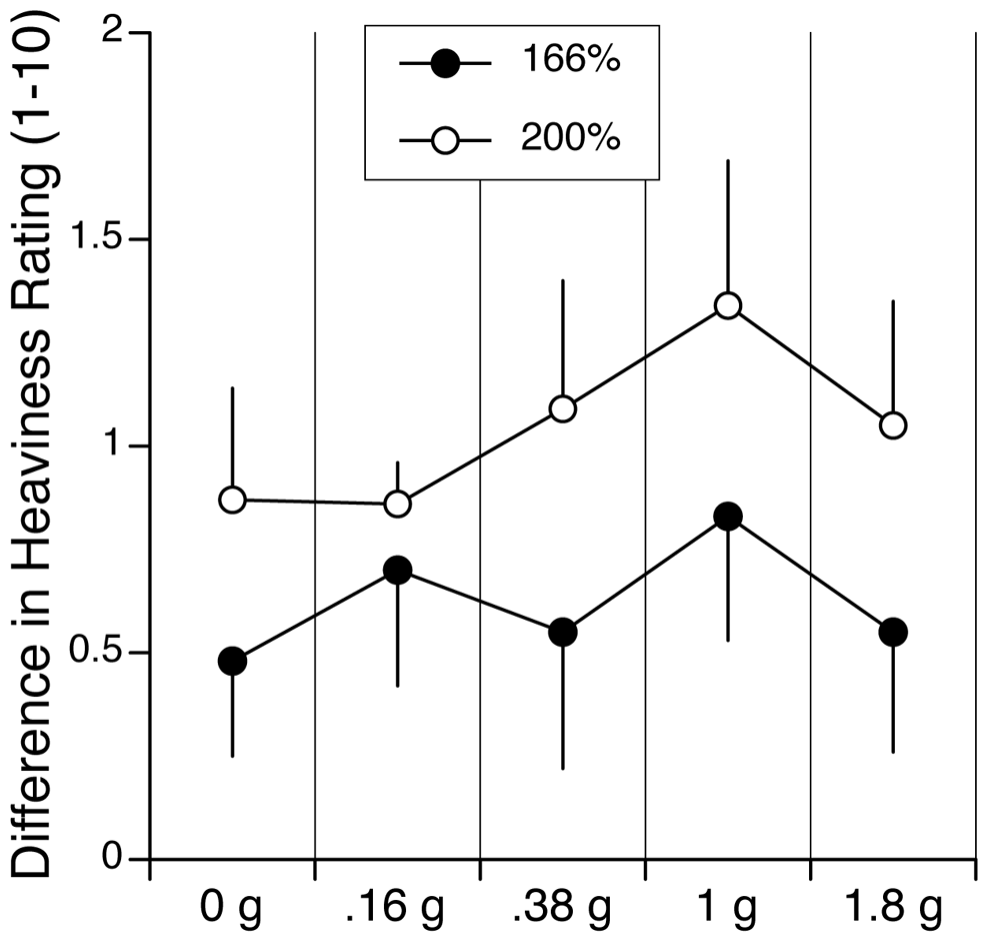
Differences in heaviness ratings between the smallest cube and the objects that were 166% and 200% larger than the smallest cube for the various gravity levels (mean and SE; N = 6 subjects in 0.16 g and 0.38 g; N = 12 subjects in zero g and 1.8 g; N = 18 subjects in one g). All objects had the same mass (1 kg). The magnitude of the size-mass illusion was larger for the taller objects at all gravity levels.

The difference in heaviness ratings between the small object and the big objects with the same mass was significantly different from zero (p<0.01) for all gravity conditions (Figure 6). This result clearly indicates that the normalized magnitude of the SMI doesn’t appear to change as a function of the gravity level.

**Figure 7:**
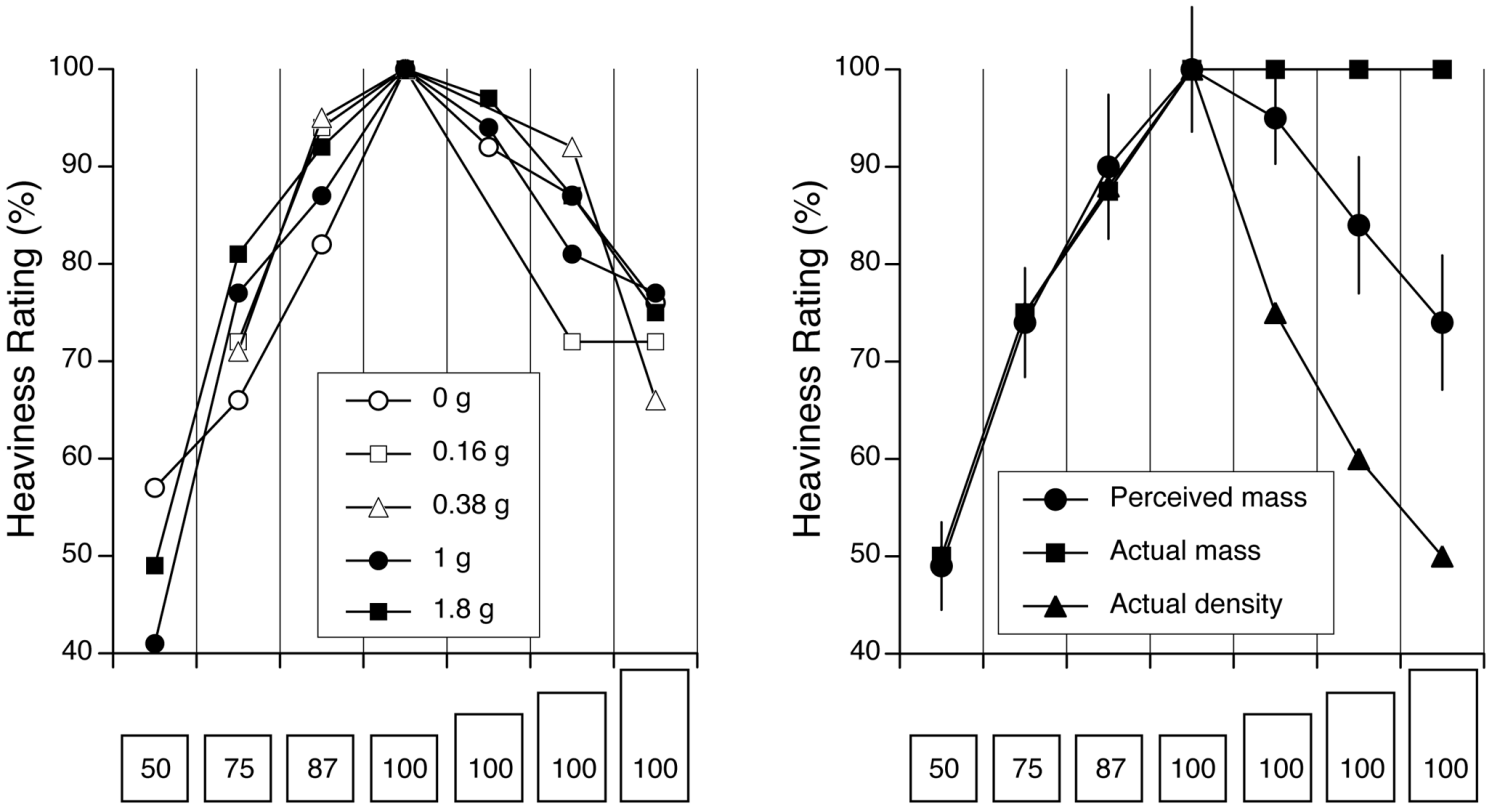
Heaviness ratings expressed in percent relative to the reference cube for the seven objects at each gravity level (mean of all trials and subjects) (A). Comparison between the perceived mass at all gravity levels combined (mean ± SD of data shown in A) and the actual mass and density of the objects (**B**).

Figure 7A shows the verbal judgments of mass for all gravity levels normalized to the reference cube. The perception of the relative mass between all seven objects was consistent across all gravity levels. For the cube-shaped objects, the subjects were very accurate with their relative mass estimates, as shown by the high correlation coefficient between perceived mass and actual mass (r^2^ = 0.93). For the objects with the same mass, when the height of the object increased by a factor of two, its perceived mass decreased by a factor of 1.3. It is also interesting to note that for the cube-shaped objects the mass estimates matched the object density, which was not the case for the objects with a different height (Figure 7B). The fact that the SMI appear to be unrelated to the density of the objects has previously been observed by Buckingham & Goodale [15].

## Discussion

The results of this study indicate that the ability to discriminate mass is affected by the gravity level. However, subjects can accurately estimate the *relative* mass between objects. In addition, when the mass of two objects is identical the large object is perceived to be lighter than the small object. The magnitude of this size-mass illusion is not affected by the gravity level. In other words, looking at the size of the objects predetermines our estimation of their relative mass, irrespective of the gravity level. These results confirm the observations made in ground-based studies using suspended objects [9], [10] or air bearing devices [11] that simulated the absence of gravitational force. In agreement with these studies and others [14], [15], [16] our findings also suggest that the role of the perceptual system is predominant on the role of the sensorimotor system for perceiving the relative mass of objects.

When lifting an object in a gravitational environment a continuous downward pressure is exerted on the hand, which must be added to the pressure produced by arm accelerations in a particular direction. Thus, both weight and inertial mass can be sensed, whereas in zero g weight cues are effectively absent. In zero g, mass can only be evaluated via inertial cues by accelerating (shaking) the objects. During these active movements, the brain must monitor the command signals to the arm muscles to distinguish between the proportions of the reactive force due to the imposed acceleration. Misperceptions could occur because of imperfect monitoring of command signals; incorrect scaling of afferent signals for muscle, joint and pressure receptors; or incorrect expectations of the relation between efferent and afferent signals. The loss of weight information in zero g is equivalent to a reduction in sensory information: no information is gained while the object is static or moving at constant velocity, but only during periods of positive or negative acceleration. In zero g, proprioceptive information is present only when the reactive force from the mass of the object stimulates the pressure receptors in the hand. It was argued that the mass discrimination impairment in zero g was due to the loss of the pressure (weight) exerted by the object on the subject’s hand, leading to rely only on the ratio of force acceleration (mass) produced by shaking movements [7].

Previous studies have shown that the human ability to discriminate mass is deteriorated in non-terrestrial environments. A study on Spacelab-1 (STS-9) and STS-61 used metallic balls of same size but different masses (approximately 50 gr) [12]. Five astronauts were instructed to pick up a numbered ball from a container, shake it free-floating by hand with amplitude 20–40 cm and return it to the container. A second ball was then picked up and it too was shaken from side to side. After returning the second ball to its slot, the astronaut then stated which ball felt “heavier”. Each subject was instructed that one ball would always feel heavier and to guess if unable to tell a difference (forced choice). Results showed that objects in zero g were perceived to have a mass reduced by a factor of 1.8 compared to the same objects in one g. In our study the verbal judgments of mass in zero g were reduced by a factor of 1.6 compared to one g. This difference between may be due to the fact that in orbit the objects were shaken in free space, while in our study in zero g the objects were in contact with a support surface. The residual frictional forces with this support surface could be at the origin of larger estimates of mass.

Ross repeated her experiment in parabolic flight with more volunteers. During the zero g periods of parabolic flight, objects were perceived to be about half of their mass in one g. The authors reported that the greater impairment in mass discrimination seen in parabolic flight could have been due to the shorter time available for adaptation (compared to spaceflight), to “fluctuating” 0 g levels in the aircraft, or to suboptimal shaking techniques by most of the subjects who had received less training for that task than the astronauts [7], [13]. We have proposed to repeat our experiment on board the International Space Station to evaluate the differential effect of these factors on the results.

Ross et al. [17] have proposed that the impaired mass discrimination in orbit was due to an incomplete adaptation to the altered gravity level during seven days in space. The STS-9 and STS-61 astronauts also reported that their bodies and other objects felt to be extra heavy post-flight. Anecdotal reports by astronauts and cosmonauts suggest that both other objects and their own bodies feel “too light” in orbit and “too heavy” on return to Earth. Cosmonauts returning from short space missions estimated objects as feeling 2–3 times their normal weights immediately post-flight, although the effect dissipated rapidly [18]. These observations make it likely that crewmembers adapt to the loss of weight in orbit and experience an after-effect of heaviness on return to Earth [19].

Generally, when a subject first experiences an altered gravity environment, his arm movements are likely to be inappropriate. On Earth, the arm is moved up or held in a raised position against the gravity force, whereas in zero g it can be moved in any axis without encountering any opposing force [20], [21]. Therefore, subjects tend to overreach in zero g and to under-reach at high g and on return to one g after spaceflight. Fine motor skills are likely to be upset until adaptation is complete, and distortions of weight or mass perception are also likely to occur [22].

Impairment in mass perception has been also observed in centrifuges generating hypergravity of 2 g and 4 g. Darwood et al. [23] used 30 egg-shaped objects in a human-rated centrifuge. Fifteen of the objects were 100 gr and three each of the other 15 were 105, 110, 115, 120, and 125 gr, respectively. Mass discrimination, evaluated by the method used by Ross et al. [12] described above, deteriorated in 2 g and 4 g during either 5-min continuous exposure or 30-sec alternating exposure. The reason the mass discrimination deteriorated was presumably because the weight of the subject’s arm also changed as a function of increasing gravity level. It is likely that the change in arm weight was responsible for the perceived change in object mass. Mass discrimination deteriorated in hypergravity as a function of increasing gravity level. The deterioration observed at four g was similar to that seen at zero g [24]. These results are in agreement with our study. We found that the verbal judgments of mass for the objects increased by a factor of about 1.3 in 1.8 g compared to one g. So the deterioration at 1.8 g was less than that seen in zero g.

One interesting question is why is the perceived mass different in various gravitational environments? Our results show that arm acceleration and mass estimates both increase with gravity level. The increase in arm acceleration is presumably due to the increase in contact force between the object and the lap desk when gravity increases. The higher the gravity level, the larger the force needed to move the object from side to side, and therefore the larger the estimate of mass. When normalizing the responses, the results also clearly indicate that subjects were able to accurately estimate the relative mass between objects for all gravity levels.

Unfortunately, because all the objects had the same size in Ross and Albery’s studies above, the role of cognitive system in learning the normal relations between the appearance of objects and their weight could not be tested in zero g and hypergravity. Our study indicates that when the mass of two objects is identical the large object is perceived to be lighter than the small object. The magnitude of this SMI is not affected by the gravity level. However, the changes in arm accelerations due to the gravity level were independent of the object’s features, i.e. mass and size. Consequently, for the objects with the same mass and a different height we did not observe a sensorimotor version of the perceptual size-mass illusion, i.e. the arm acceleration was not smaller for the large object compared to the small object. The results of previous studies showing that, with the repetition of the trials, there is no adaptation of the SMI but there is an adaptation of the finger grip force [14], [15], [16] are in agreement with our data showing that SMI is not systematically related to arm acceleration. This finding confirms that the perceptual cognitive system plays a larger role in the SMI that the sensorimotor system.

In conclusion, in agreement with previous studies, our results show that gravity plays an essential role in *absolute* mass discrimination. However, just as humans are more reliable in estimating the relative weights between objects than their absolute weights [25], they are also more reliable in estimating the *relative* mass between objects than their absolute mass. In addition, the accuracy of these relative mass estimates between objects is not affected by gravity. Consequently, it is expected that astronauts on the Moon or Mars should have no difficulty for estimating the relative mass between objects, provided that they keep their eyes closed. When they look at these objects, however, their perceptual cognitive system may induce a size-mass illusion. So, just like here on Earth, on the Moon or Mars one kg of feathers would feel lighter than one kg of lead!
